# Administration of Chinpi, a Component of the Herbal Medicine Ninjin-Youei-To, Reverses Age-Induced Demyelination

**DOI:** 10.1093/ecam/neq001

**Published:** 2011-06-05

**Authors:** Nanako Sato, Chika Seiwa, Michihiro Uruse, Masahiro Yamamoto, Kayoko Tanaka, Takuya Kawakita, Yasuhiro Komatsu, Akio Yasukawa, Masakatsu Takao, Chiaki Kudo, Atsuhiko Hasegawa, Atushi Ishige, Kenji Watanabe, Hiroaki Asou

**Affiliations:** ^1^Department of Neuroglia Cell Biology, Tokyo Metropolitan Institute of Gerontology, 35-2 Sakae-cho, Itabashi-ku, Tokyo 173-0015, Japan; ^2^Center for Kampo Medicine, Keio University School of Medicine, 35 Shinanomachi, Shinjyuku-ku, Tokyo 160-8582, Japan; ^3^Tsumura Laboratory, Tsumura & Co., 3586 Yoshiwara, Ami-machi, Inashiki-gun, Ibaraki 300-1192, Japan; ^4^Biomedical Research Center, Division of Morphological Science, Saitama Medical School, 38 Morohongo, Iruma-gun, Saitama, 350-0495, Japan; ^5^Kracie Pharmaceutical, Ltd., 1-8-63 Tenmanbashi, Kita-ku, Osaka 530-0042, Japan; ^6^Sun R&D Institute for Natural Medicine Co. Inc., 3-12-6 Ginza, Chuo-ku, Tokyo 104-0061, Japan; ^7^Nishiogi Animal Hospital, 4-4-5 Nishiogikita, Suginami-ku, Tokyo 167-0042, Japan; ^8^NHK Enterprises, Inc., 4-14 Kamiyama-cho, Shibuya-ku, Tokyo 150-0047, Japan; ^9^Kudou Chiaki Hospital, 1-23-10 Omori-kita, Ota-ku, Tokyo 143-0016, Japan; ^10^Department of Pathobiology, Nihon University School of Veterinary Medicine, 1866 Kamei-cho, Fujisawa-shi, Kanagawa 252-8510, Japan

## Abstract

The disruption of myelin causes severe neurological diseases. An understanding of the mechanism of myelination and remyelination is essential for the development of therapeutic strategies for demyelination diseases. Our previous findings indicated that the FcR*γ*/Fyn cascade is a potential therapeutic target for remyelination caused by the Chinese/Japanese traditional herbal (Kampo) medicine ninjin'youeito (Ninjin-youei-to, NYT), which is a hot-water extract made from 12 medicinal herbs. To identify which constituents of NYT are involved in the reversal of demyelination and to examine the potential therapeutic effect, we tested several of the chemical constituents of NYT. Here, we report that Chinpi, a constituent of NYT, upregulates the FcR*γ*/Fyn signaling cascade resulting in a potentially therapeutic effect against age-induced demyelination. In addition, we observed that phosphorylated (activated) FcR*γ*/Fyn upregulated the expression of the 21.5 kDa isoform of myelin basic protein, inducing rapid morphological differentiation, when oligodendrocyte precursor cells (OPCs) were cultured in the presence of hesperidin and/or narirutin (the major active constituents of Chinpi). These results suggest that hesperidin and narirutin participate in the FcR*γ*/Fyn signaling pathway in OPCs causing these cells to differentiate into myelinating oligodendrocytes.

## 1. Introduction

The myelin sheath of the central nervous system (CNS) is formed by oligodendrocytes wrapping layer upon layer of their own membranes around axons in a tight spiral, forming an electrically insulating sheath around each axon. The destruction of this myelin sheath in the CNS causes severe neurological disorders [1–3]. Congenital dysmyelination causes mental retardation in children, while demyelination later in life results in a decline in cognitive function. Elucidating the mechanism of demyelination/remyelination should provide new insight for future therapeutic strategies against dysmyelinating or demyelinating human diseases such as multiple sclerosis (MS), although no therapy has yet been established to prevent such disease progression [4–6]. Although the reasons for the eventual failure of remyelination (demyelinating diseases) are unknown, the presence of oligodendrocyte precursor cells (OPCs) and immature oligodendrocytes in some non-repairing lesions suggests that these cells may fail to receive the necessary differentiation signals [7–10]. Further elucidation of the processes involved in OPC maturation may lead to new therapeutic strategies for MS and other related diseases [11].

We previously demonstrated that the level of phosphorylated myelin basic proteins (P-MBPs), especially the 21.5 kDa isoform, but not the level of total MBPs was markedly and inversely correlated with demyelination induced by aging and/or the neurotoxin cuprizone and that the low levels of 21.5 kDa P-MBP in demyelinated brain recovered significantly after treatment with a Japanese herbal medicine, Ninjin-youei-to (NYT) [12]. We hypothesized, therefore, that the 21.5 kDa P-MBP level might reflect the status of myelination, indicating the levels of demyelination or remyelination.

NYT is a hot-water extract prepared from a mixture of several medicinal plants. A high-performance liquid chromatography (HPLC) analysis performed in our previously report [12] confirmed that the ingredients of the NYT water-soluble extracts yielded a variety of peaks corresponding to the chemical constituents of NYT: paeoniflorin and albiflorin (from *Peoniae radix*, [Shakuyaku]); liquiritin, liquiritin apioside, isoliquiritin apioside, isoliquiritin and glycyrrhizin (from *Glyccyrrhizae radix*, [Kanzo]); narirutin and hesperidin (from *Aurantii nobilis pericarpium*, [Chinpi]); cinnamaldehyde and cinnamic acid (from *Cinnamomi cortex*, [Keihi]); and 3,6′-disinapolysucrose (from *Polygalae radix*, [Onji]). These peaks appeared at the appropriate retention times and exhibited a dose-dependent correlation with the concentration of the NYT solution. Surprisingly, the HPLC analysis was particularly sensitive for detecting narirutin and hesperidin, even at the lowest NYT concentrations.

NYT has not yet been approved as a therapeutic drug for the recovery of age-induced demyelination (data not shown). To further characterize the major active constituents of NYT in the reversal of age-induced demyelination, the constituents of NYT extract (made from 12 compounds) were grouped into four mixtures based on the results of a 3D-HPLC analysis of NYT. The purpose of this study was to investigate the accessibility of these effective constituents of NYT to areas of demyelinated brain after oral administration and to determine the cellular and molecular mechanism underlying the biological efficacy of the major active constituents of NYT.

## 2. Materials and Methods

### 2.1. Mice

Twenty-six-month-old C57BL/6 mice were supplied by the Department of Animal Science, Tokyo Metropolitan Institute of Gerontology. Double-deficient (dKO) adult mice were obtained by crossing FcR*γ*-deficient [13] and Fyn-deficient [14] mice, as described previously. All the mice, including the mutants, were from a C57BL/6 background strain.

### 2.2. Animal Experiments

All the procedures used in this study were in accordance with the guidelines of our institutional animal care and use committee of Tokyo Metropolitan Institute of Gerontology (approval No. 08045) and Keio University School of Medicine (approval No. 08071).

### 2.3. Treatment with Ingredients Contained in NYT Extract

NYT is a spray-dried extract of a mixture of 12 raw medicinal plants and is manufactured by Kracie Pharma, Ltd. (Tokyo) under strict scientific and quality control protocols; it has been approved for medical use by the Ministry of Health, Labour, and Welfare of Japan. The compositions of the plants and the 3D-HPLC profile of NYT have been previously reported [12, 15]. Here, we examined four groups of NYT constituents. Using an age-induced demyelination model, 1% of each constitutive herb was administered in the drinking water of the animals for 2 months prior to sacrifice. The constitutive herb mixtures were as follows: (i) mixture A (*Cinnamomi cortex* [Keihi], 37.5 g; *Polygalae radix* [Onji], 30 g; *Peoniae radix* [Syakuyaku], 30 g; *Aurantii nobilis pericarpium* [Chinpi], 30 g; and *Glyccyrrhizae radix* [Kanzo], 15 g), (ii) mixture B (*Cinnamomi cortex* [Keihi], 75 g; and *Peoniae radix* [Syakuyaku], 60 g), (iii) mixture C (*Polygalae radix* [Onji], 80 g; and *Glyccyrrhizae radix* [Kanzo], 15 g) and (iv) mixture D (*Aurantii nobilis pericarpium* [Chinpi], 50 g). A previously described method was used to perform 3D-HPLC analyses of these NYT constituents [12, 15]. Supplementary Figure 1 shows the results of the 3D-HPLC analysis for Chinpi.

### 2.4. Tissue Preparation and Histopathological Analysis

Mice (4-months old) were perfused with 4% paraformaldehyde and their brains were fixed overnight in an acid alcohol solution (95% ethanol/5% acetic acid, v/v). The brain tissues were then embedded in paraffin and sectioned into 10 *μ*m thick slices. The sections were then mounted and stained for myelin using Luxol Fast Blue (LFB: Acros Organics, Fair Lawn, NJ, USA) solution (0.1% LFB in 95% ethanol containing 0.05% acetic acid [Wako]; stained overnight at 60°C). Toluidine blue (Wako) staining was performed according to the manufacturer's protocol (Wako).

### 2.5. Electron Microscopy

Each cerebrum was fixed with 2.5% glutaraldehyde and then post-fixed with 1% OsO_4_. After dehydration in ethanol, the specimens were embedded in Quetol 812 (Nisshin EM, Tokyo, Japan). Ultrathin sections stained with 2% uranylacetate and lead solution were observed using a JOEL 100C electron microscope (JOEL, Tokyo, Japan), as described elsewhere [12]. For the G-ratio measurement (the ratio of the axon diameter to the diameter of the axon plus the surrounding myelin), at least three mice per group were used. Eight to ten microphotographs of the corpus callosum (coronal) at midline and at a high magnification (×5000, ×10 000) were taken for each mouse, and the G-ratios of at least 50 axons were measured. Axons with aberrant morphology, such as myelin reduplication, abnormal splitting of the myelin sheath, vacuolization of the myelin lamellae, and myelin balloon formation, were excluded because such aberrant myelin morphology makes it impossible to evaluate the degree of myelination accurately. For example, the extraordinarily large myelin sheath of axons with an abnormal morphology arising from the repetition of unaccomplished remyelination should not be estimated in the same manner as the myelin sheaths of normally developed myelinated axons. An additional quantitative study was also undertaken to assess the number of myelinated fibers per 400 *μ*m^2^ using three mice per group (Figure 2).

### 2.6. Preparation of Myelin

Myelin was prepared as described previously [12]. Briefly, the cerebrum (0.2 g) was homogenized using a Teflon homogenizer in 20 volumes (w/v) of 0.32 M sucrose and the resulting homogenate was layered over 0.85 M sucrose. After centrifugation at 25 000 r.p.m. for 30 min, the layer of crude myelin layers was re-suspended in water by homogenization and then washed by repeated centrifugation. The homogenate was once again centrifuged on a 0.85 M sucrose bed, and the purified myelin was removed.

### 2.7. Western Blot Analysis

The western blot analysis was performed as described previously [12]. The membranes were probed with polyclonal antibodies to FcR*γ* [13], Fyn (Dr K. Senzaki, National Institute for Physiological Science, Japan) and MBP (Nichirei, Tokyo, Japan), and monoclonal antibodies to phosphotyrosine (PY20; Calbiochem), phosphorylated MBP (Upstate Biotechnology), actin (Sigma) and CNPase (Sigma). Quantification of the bands was performed using densitometry and Image-J Software. Differences in the amounts of loaded proteins among the lanes in a single experiment were normalized according to the amount of actin that was loaded.

### 2.8. OPC Culture and Stimulation

Mouse OPCs were obtained from the cerebral hemispheres of embryonic day 17 mice. We cultured the mouse OPCs as previously described [16]. For the stimulation experiments, the OPCs were seeded at a density of 1 × 10^6^ cells per plate and each mixture of NYT constituent or ingredient compounds was added so that the final concentration of each compound was 10 ng/mL. After 48 h, the cells were harvested. The cell lysates were prepared and SDS-PAGE and immunocytochemistry were performed as previously described [16].

### 2.9. Immunoprecipitation and Immunoblotting

Cultured OPCs were lysed in a solution containing 50 mM Tris–HCl (pH 7.4), 1% NP-40, 0.25% sodium deoxycholate, 150 mM NaCl, 1 mL EDTA-2Na, 1 mM Na_3_VO_4_, 1 mM NaF, 1 mM PMSF, 2 *μ*g/mL aprotinin and 4 *μ*g/mL leupeptin (Calbiochem, LaJolla, CA, USA). The lysates were then purified by centrifugation at 13 000 g. The protein concentration of each lysate was measured using a micro BCA protein assay reagent (PIERCE, Rockford, IL, USA). For immunoprecipitation, the lysates were incubated overnight at 4°C with anti-FcR*γ* and anti-Fyn polyclonal antibodies [11] and anti-phosphotyrosine (PY-20) monoclonal antibody, followed by incubation for 2 h at room temperature (RT) on a rotating shaker with protein G sepharose (Amersham Biosciences, Uppsala, Sweden). The samples were washed three times with a solution containing 10 mM Tris (pH 7.4), 150 mM NaCl and 0.05% Tween-20. The immunoprecipitated material was centrifuged at 12 000 g for 15 min, and the resulting pellet was washed once in washing buffer without Tween-20, then resolved using SDS-PAGE (4–20% gradient polyacrylamide gel) for 2 h. The proteins were then electroblotted using a semi-dry transfer onto an Immobilon PVDF membrane (Millipore, Bedford, MA, USA). The membranes were blocked with 5% skimmed milk (Difco Laboratories, Detroit, MA, USA) or 3% bovine serum albumin (for the phosphor-antibodies) in Tris-buffered saline (TBS; pH 7.6) for 1 h at RT, and then the blots were incubated with several primary antibodies for 2 h at RT. Next, the membranes were washed three times for 10 min each using 1% skimmed milk in TBS, were incubated with alkaline phosphatase-conjugated goat anti-mouse or anti-rabbit IgG (diluted 1 : 800) for 2 h at RT, and were developed using nitro blue tetrazolium (NBT) and 15-bromo-4-chloro-3-indolyl-phosphate (BCIP) solution (both from Sigma, St Louis, MO, USA). The signal bands were semi-quantified using the Kodak Electrophoresis Documentation and Analysis System (EDAs) 290 (Eastman Kodak Company, Rochester, NY, USA).

### 2.10. Statistics

Morphological data are shown as the mean ± SEM. Differences among groups were analyzed using the Student *t*-test with a Bonferroni correction for multiple comparisons. A value of *P* < .05 was considered statistically significant. Differences between NYT-treated samples and their untreated counterparts were assessed using the Welch *t*-test with a Bonferroni correction.

## 3. Results

### 3.1. Chinpi Ameliorates Age-Induced Demyelination via the FcR*γ*/Fyn-MBP Cascade

To elucidate the active constituent herbs of NYT, which is a hot-water extract of a combination of 12 medicinal plants, the constituents of NYT were grouped into four mixtures and these mixtures were administered to elderly mice for 2 months. After the treatment period, the total MBP levels in the brains of the mice were not significantly different among the groups (mixtures A, B, C and D).

**Figure 1:**
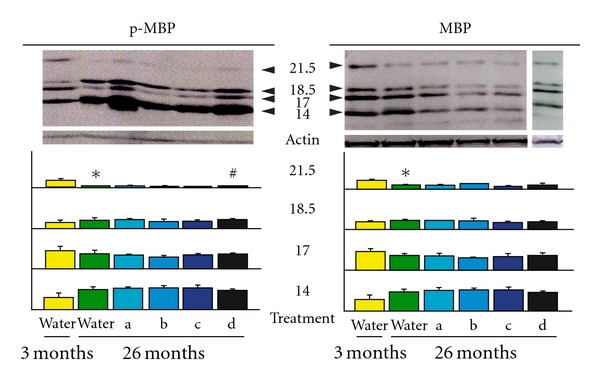
The 21.5 kDa phosphorylated MBP (P-MBP) levels were closely related to the myelination status. Aging-induced demyelination and its improvement by different constituents of NYT were accompanied by the specific disappearance and reappearance of 21.5 kDa P-MBP protein, respectively. The levels of the 21.5-, 18.5-, 17- and 14-kDa isoforms (21.5, 18.5, 17 and 14) of total MBPs and P-MBP were quantified using an immunoblot analysis of isolated myelin sheaths. Because the basal levels of P-MBPs and MBPs fluctuate during aging, the quantitative ratio of the four isoforms was calculated for each age. The quantification and statistical analyses were performed as described in the ‘Materials and Methods' section. Data represent the mean ± SEM (*n* = 3). *P < .005 versus untreated (3 months of water alone), ^#^ *P* < .05 versus untreated. a: Mixture A (Keihi, Onji, Shakuyaku Kazo, Chinpi); b: Mixture B (Keihi, Shakuyaku); c: Mixture C (Onji, Kanzo); d: Mixture D (Chinpi).

For our initial evaluation of the ameliorating efficacy of the mixtures, an immunoblot analysis of the phosphorylated and total MBPs was performed. In a previous study [12], we demonstrated that the myelination status was closely related to the levels of phosphorylated MBPs, especially to that of the 21.5 kDa isoform. The specific disappearance and reappearance of 21.5 kDa P-MBP occurred in parallel with demyelination and remyelination in cuprizone- and aging-induced demyelination models, while the total MBP levels were not significantly correlated with these processes. In the present study as well, the amount of phosphorylated 21.5 kDa MBP (21.5 kDa P-MBP), which decreases as a result of age-induced demyelination, was restored after treatment with mixtures A and D. Mixture D was particularly effective for restoring the 21.5 kDa P-MBP level (Figure 1). These results are consistent with those of our previous report, which showed that NYT promotes remyelination as shown by the reappearance of 21.5 kDa P-MBP after cuprozone-induced or age-induced demyelination [12].

**Figure 2:**
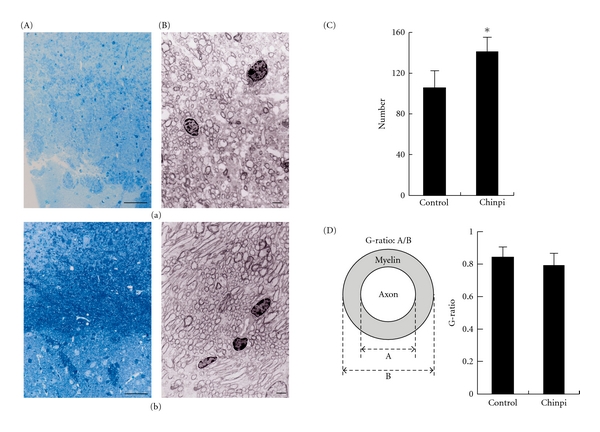
Suppression of age-induced demyelination by Chinpi. (A) Toluidine blue staining of cross-sections of the corpus callosum shows age-induced demyelination in control mice (untreated) (a) and the reversal of age-induced demyelination in 26-month-old mice after treatment with Chinpi for 2 months (b). (B) Electron micrographs of cross-sections of the corpus callosum show age-induced demyelination in 26-month old (a) mice. The myelination status was greatly improved after Chinpi treatment for 2 months (b). (C) The density of myelinated fibers per 400 *μ*m^2^ was lower in untreated 26-month-old mice, and treatment with Chinpi led to a significant recovery in the myelinated fiber density. The number of myelinated fibers in each mouse was counted using 4–6 electron micrographs and was averaged. Data represent the mean ± SEM (*n* = 3); *P < .005. (D) The G-ratio, an indicator of demyelination defined as the ratio of the diameter of the axon to the diameter of the axon plus the surrounding myelin (the formula is shown in the insert), shows that the increased G-ratio in the elderly mice was abrogated by Chinpi treatment. The number of measured axons determined from 3 to 5 mice per group was 54–109 using 8–10 electron micrographs per mouse. Data represent the mean ± SEM. *P* > .01 versus control.

We next used toluidine blue staining and electron microscopy of the corpus callosum to examine the demyelination/remyelination status in elderly brain specimens after treatment with Chinpi. Consistent with previous findings obtained using a diet containing 1% NYT, feeding mice with a diet containing 1% Chinpi for 2 months markedly ameliorated the age-induced demyelination observed in 26-month-old wild-type mice. While the brains of untreated, elderly mice showed many abnormalities in the myelin sheaths, such as myelin balloon formatiozn, splitting of the myelin sheath, vacuolization of myelin lamellae, paranodal retraction and abnormal tight junctions (abnormalities that are rarely observed in younger mice); a marked reduction in abnormal histological findings was observed in the brains of elderly mice treated with 1% Chinpi in their drinking water for 2 months (Figure 2). Furthermore, the density of myelinated fibers per 400 *μ*m^2^, which had decreased in the 26-month-old untreated mice, was restored after treatment with Chinpi for 2 months (Figure 2). The thin and shortened myelin sheaths that are characteristic of remyelination can be readily identified using electron microscopy to calculate the G-ratio (the ratio of the axon diameter to the diameter of the axon plus the surrounding myelin). In the elderly mice that were treated with Chinpi for 2 months, the presence of many small myelinated axons, which were presumed to have arisen from remyelination, was observed (Figure 2). The present data are consistent with the previously reported finding that the myelination status was restored after 3 days of treatment with NYT in a demyelination model induced by 5 weeks of cuprizone treatment [12]. These results suggest that NYT promotes remyelination, rather than preventing demyelination.

**Figure 3:**
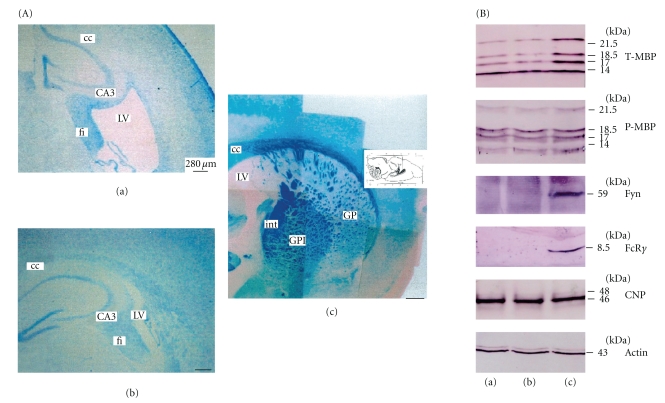
Chinpi had no therapeutic efficacy on demyelination in FcR*γ*/Fyn double-deficient mice (dKO). (A) Luxol Fast Blue (LFB) staining of cross-sections of the corpus callosum from untreated dKO mice (a), dKO mice after treatment with Chinpi for 2 month (b) and 3-month-old control mice (c). cc, corpus callosum; LV, lateral ventricle; GPI, globus pallidus (lateral segment); CP, caudate putamen; fi, fimbria; CA3, field CA3 in the hippocampus; int, internal capsule. (B) Immunoblot analysis of MBP and P-MBP levels from myelin in FcR*γ*/Fyn double-deficient (dKO) mice after treatment with Chinpi for 2 months. (a) Untreated control, (b) Chinpi treatment, (c) Normal control mice (3-months old).

Our previous findings also suggested that the FcR*γ*/Fyn-MBP cascade might be a potential therapeutic target of NYT in the pathway leading to remyelination, since NYT promoted myelination in wild-type mice but not in FcR*γ*/Fyn dKO mice [12]. To further characterize the efficacy of the NYT constituents, we examined the efficacy of the above-mentioned mixtures of NYT constituents on the activation of the FcR*γ*/Fyn cascade, which leads to myelination, using wild-type and FcR*γ*/Fyn dKO mice. In particular, since the first part of this study focused on the recovery of the 21.5 kDa P-MBP level after treatment with mixture D (Chinpi), we examined the efficacy of Chinpi on FcR*γ*/Fyn dKO mice. An immunoblot analysis of FcR*γ*, Fyn and MBP revealed that Chinpi apparently upregulated the expressions of Fyn and FcR*γ* in wild-type mice treated with either mixture A or D (data not shown) but that the 21.5 kDa P-MBP level was not affected by treatment with Chinpi in dKO mice nor was the expression of the adult myelin marker CNPase significantly different after Chinpi treatment (Figure 3).

To gain new insight into the molecular mechanisms responsible for the efficacy of Chinpi on the reversal of demyelination, we also examined the efficacy of Chinpi treatment in FcR*γ*/Fyn dKO mice. These mutant mice exhibit almost no change in phosphorylated/dephosphorylated MBP levels during cuprizone-induced demyelination, and previous observations have shown no signs of remyelination arising from the reappearance of phosphorylated 21.5 kDa MBP and no electron microscopy evidence of remyelinated fibers, even after treatment with NYT for 2 months [12]. As expected, treatment with Chinpi had no effect on the levels of P-MBPs, including the 21.5 kDa isoform (Figure 3), in the dKO mice, even though these levels were increased by treatment with mixtures A and D (Chinpi) in the wild-type mice (data not shown).

### 3.2. Hesperidin and Narirutin Promote OPC Differentiation via the Upregulation of 21.5 kDa P-MBP

**Figure 4:**
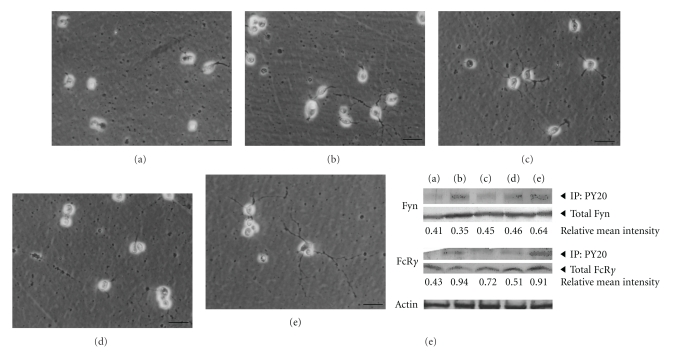
NYT constituents enhance the activation of FcR*γ*-Fyn signaling in OPC cultures. The mixtures of NYT constituents were added to the OPC cultures at a final concentration of 10 ng/mL for each constituent. After 48 h, the cell lysates were immunoprecipitated using anti-PY20 antibody and detected using anti-Fyn or anti-FcR*γ* antibody. The blots were probed with antibodies against anti-PY20 antibody and re-probed with total Fyn or total FcR*γ* antibodies to normalize the mean intensity of phosphorylation. The phosphorylation of FcR*γ* was greater in cells treated with mixtures A or D. (a) Untreated control OPC culture, (b) mixture A (Keihi, Onji, Shakuyaku, Chinpi, Kanzo), (c) mixture B (Keihi, Shakuyaku), (d) mixture C (Onji, Kanzo), (e) mixture D (Chinpi).

**Figure 5:**
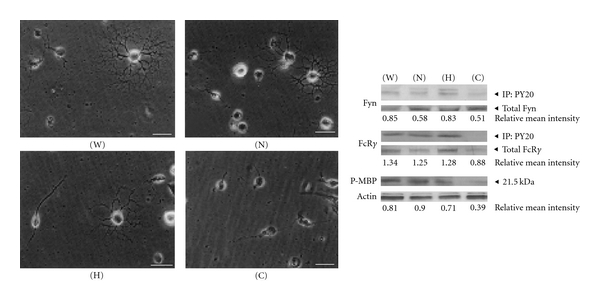
Chinpi's active constituents, hesperidin and narirutin, activate the FcR*γ*/Fyn signaling cascade and increase the level of 21.5 kDa P-MBP in OPC cultures. Mixtures of NYT constituent were added to the OPC cultures at a final concentration of 10 ng/mL for each constituent. After 48 h, the cell lysates were immunoprecipitated using anti-PY20 antibody and detected using anti-Fyn or anti-FcR*γ* antibodies. The OPCs that had been incubated with hesperidin and/or narirutin showed signs of differentiation. The cells also showed a marked increase in the level of 21.5 kDa P-MBP. (N) narirutin; (H) hesperidin (Both are major constituents of Chinpi; see Supplementary Figure 1); (W) narirutin plus hesperidin; (C) untreated control OPC cultures.

OPCs accumulate during the depletion of mature oligodendrocytes or demyelination, and OPCs located near lesions begin to differentiate and may actively participate in remyelination. Thus, the stimulation of OPCs could serve as a valuable tool for repairing functional damage caused by demyelination. Therefore, we next tested whether FcR*γ*/Fyn signaling is stimulated by NYT constituents in OPC culture. Although myelin compaction cannot, at present, be modeled in an *in vitro* experimental system, FcR*γ*/Fyn signaling is known to play an extraordinarily important role in OPC differentiation/myelination [11].  Figure 4 shows several constituents of NYT and their effects on FcR*γ*/Fyn signaling *in vitro*; in particular, mixtures A (Keihi, Onji, Shakuyaku, Kanzo and Chinpi) and D (Chinpi) dramatically activated FcR*γ*/Fyn signaling. We also investigated the molecular and cellular mechanisms underlying the potential efficacy of hesperidin and narirutin, chemical constituents of Chinpi, on FcR*γ*/Fyn signaling using OPC cultures (Figure 5), with results that were similar to those obtained using mixture A and D (Figure 4). Thus, both hesperidin and narirutin, which are active compounds of Chinpi, appear to activate the FcR*γ*/Fyn signaling pathway leading to an increase in the 21.5 kDa P-MBP level in OPC cultures, which in turn activates modest morphological differentiation (Figure 5). This observation is consistent with previous findings showing that the FcR*γ*/Fyn level is upregulated by several folds during OPC differentiation [11].

## 4. Discussion

The results of the present article support and extend our previous findings showing a beneficial efficacy of NYT treatment on aged-induced demyelination. Specifically, we have shown that the recovery of demyelination in Chinpi-treated elderly mice is associated with an improvement in the preservation of myelinated fibers, as shown using an electron microscopy analysis (Figure 2). These findings are consistent with those of previous studies showing that recovery from demyelination in cuprizone-treated and elderly mice is achieved after the administration of NYT and suggesting that the restoration of FcR*γ*/Fyn signaling is involved in remyelination [12].

In general, each herbal constituent of a Kampo medicine, such as NYT, is thought to play a particular role [17, 18], with the various constituents working together to provide the desired result. To further characterize the ameliorative efficacy of NYT on demyelinated brain, we investigated the efficacy of various mixtures of NYT constituents at concentrations comparable to those achieved when NYT is administered in drinking water. In this study, we succeeded in further isolating the active constituents of NYT. Specifically, we showed that 2 months of treatment with an orally administered hot-water extract of Chinpi (*Aurantii nobilis pericarpium*) reversed age-induced demyelination in mice (Figure 2). Moreover, this study also showed that the FcR*γ*/Fyn signaling cascade plays a crucial and presumably indispensable role in the compaction of the myelin sheath, since FcR*γ*/Fyn double-deficient (dKO) mice could not recover from demyelination even after the administration of Chinpi for 2 months (Figure 3). These findings suggest that Chinpi may restore the FcR*γ*/Fyn cascade, leading to its therapeutic efficacy. Indeed, both narirutin and hesperidin, the active constituents of Chinpi, are capable of activating FcR*γ*-Fyn signaling (Figure 5). In addition, narirutin and hesperidin dramatically increased the level of the 21.5 kDa P-MBP isoform, the regulation of which is known to be correlated with demyelination/remyelination [12]. This effect is most likely achieved by hesperidin and narirutin activating protein phosphorylation via an effector kinase of FcR*γ*/Fyn.

The consumption of dietary flavonoids has been reportedly associated with a reduced risk of neurodegenerative diseases [19–21]. Hesperidin and narirutin are citrus flavonoids that are known to have a wide variety of pharmacological activities [22, 23]. The mechanisms responsible for their various actions have not yet been elucidated. Although the bioavailability of the hesperidin and narirutin contained in NYT is uncertain, several studies have suggested that orally administered citrus peel extract or citrus juice results in the appearance of a substantial amount of flavonoids in the plasma [24–26]. In the present study, however, we suggested that hesperidin and narirutin may act directly on the reversal of demyelination in elderly mice via an effect on the FcR*γ*/Fyn signaling cascade, resulting in substantial morphological differentiation with the upregulation of MBP, especially the 21.5 kDa P-MBP.

**Figure 6:**
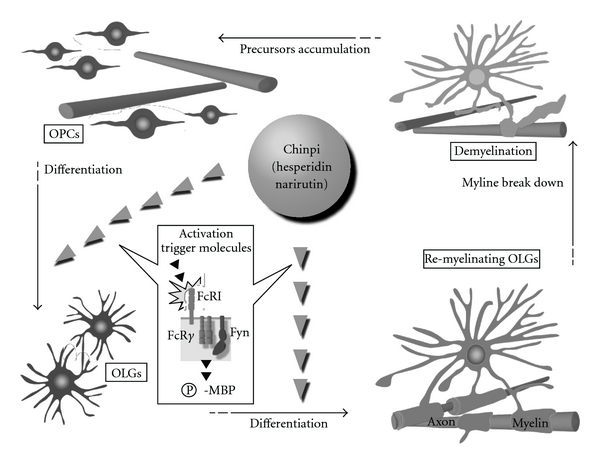
Diagram of the proposed pathways by which hesperidin and naririutin, which are active ingredients of Chinpi, stimulate the FcR*γ*/Fyn signaling cascade involved in remyelination. The activation of FcR*γ*/Fyn results in the upregulation of phospho-21.5 kDa MBP and the dramatic morphological differentiation of OPCs. OPC, oligodendrocyte precursor cells; OLGs, oligodendrocytes; P-MBP, phosphorylated myelin basic protein.

In conclusion, the present study demonstrated that Chinpi is the active ingredient in NYT responsible for the reversal of age-induced demyelination and that Chinpi stimulates the FcR*γ*/Fyn signaling cascade, resulting in the differentiation of OPCs into myelinating oligodendrocytes (Figure 6) [11]. Therefore, hesperidin and narirutin (which are the active constituents of Chinpi) may have therapeutic and physically potent effects on various neurological disorders in which demyelination plays a crucial role, including MS, dementia and schizophrenia [27].

## Supplementary Data

Supplementary data are available at *ECAM* online.

## Funding

This study was partially supported by Grants-in-Aid for Scientific Research (no. 20500348) from the Ministry of Education, Culture, Sports, Science, Science and Technology Agency of Japan (to H. A.).
